# End of an era of administering erythropoiesis stimulating agents among Veterans Administration cancer patients with chemotherapy-induced anemia

**DOI:** 10.1371/journal.pone.0234541

**Published:** 2020-06-25

**Authors:** Shamia Hoque, Brian J. Chen, Martin W. Schoen, Kenneth R. Carson, Jesse Keller, Bartlett J. Witherspoon, Kevin B. Knopf, Y. Tony Yang, Benjamin Schooley, Chadi Nabhan, Oliver Sartor, Paul R. Yarnold, Paul Ray, Laura Bobolts, William J. Hrushesky, Michael Dickson, Charles L. Bennett

**Affiliations:** 1 Department of Civil and Environmental Engineering, College of Engineering and Computing, University of South Carolina, Columbia, South Carolina, United States of America; 2 Arnold School of Public Health, University of South Carolina, Columbia, South Carolina, United States of America; 3 Department of Medicine, Saint Louis University School of Medicine, Saint Louis, Missouri, United States of America; 4 The Washington University School of Medicine and the Saint Louis VA Medical Center, St. Louis, Missouri, United States of America; 5 Medical University of South Carolina, Charleston, South Carolina, United States of America; 6 College of Pharmacy, University of South Carolina, Columbia, South Carolina, United States of America; 7 George Washington University, Washington, DC, United States of America; 8 Department of Integrated Information Technology, College of Engineering and Computing, University of South Carolina, Columbia, South Carolina, United States of America; 9 Tulane University School of Medicine, New Orleans, Louisiana, United States of America; 10 Oncology Analytics, Plantation, Florida, United States of America; 11 College of Pharmacy, Nova Southeastern University, Fort Lauderdale, Florida, United States of America; University of Colorado Denver Skaggs School of Pharmacy and Pharmaceutical Sciences, UNITED STATES

## Abstract

Erythropoisis stimulating agent (ESA) use was addressed in Food and Drug Administration (FDA) Oncology Drug Advisory Committee (ODAC) meetings between 2004 and 2008. FDA safety-focused regulatory actions occurred in 2007 and 2008. In 2007, black box warnings advised of early death and venous thromboembolism (VTE) risks with ESAs in oncology. In 2010, a Risk Evaluation Strategies (REMS) was initiated, with cancer patient consent that mortality and VTE risks were noted with ESAs. We report warnings and REMS impacts on ESA utilization among Veterans Administration (VA) cancer patients with chemotherapy-induced anemia (CIA). Data were from Veterans Affairs database (2003–2012). Epoetin and darbepoetin use were primary outcomes. Segmented linear regression was used to estimate changes in ESA use levels and trends, clinical appropriateness, and adverse events (VTEs) among chemotherapy-treated cancer patients. To estimate changes in level of drug prescription rate after policy actions, model-specific indicator variables as covariates based on specific actions were included. ESA use fell by 95% and 90% from 2005, for epoetin and darbepoetin, from 22% and 11%, respectively, to 1% and 1%, respectively, among cancer patients with CIA, respectively (p<0.01). Following REMS in 2010, mean hematocrit levels at ESA initiation decreased from 30% to 21% (p<0.01). Black box warnings preceded decreased ESA use among VA cancer patients with CIA. REMS was followed by reduced hematocrit levels at ESA initiation. Our findings contrast with privately- insured and Medicaid insured cancer patient data on chemotherapy-induced anemia where ESA use decreased to 3% to 7% by 2010–2012. By 2012, the era of ESA administration to VA to cancer patients had ended but the warnings remain relevant and significant. In 2019, oncology/hematology national guidelines (ASCO/ASH) recommend that cancer patients with chemotherapy-induced anemia should receive ESAs or red blood cell transfusions after risk-benefit evaluation.

## Introduction

Prescription drugs treat diseases and improve patients’ quality of life. However, no drug is completely safe [1,2]. Food and Drug Administration (FDA) balances risks and benefits by mandating manufacturer disclosure via warnings on product labels. Since 2007, FDA started requiring manufacturers to implement safety efforts, including consent via Risk Evaluation and Monitoring Strategies (REMS) [3–7]. A meta-analysis that public health advisories often led to decreased drug use and fleeting increases in drug monitoring [8]. No analyses report on use changes following REMS-mandated consent processes. Public health advisories impact incident, but not prevalent use [9,10]. Boxed warnings are associated with utilization reductions, but substitution between classes occurs [11–17]. Empirical evidence shows that boxed warnings have mixed results in leading to lower drug utilization. There is a need to identify factors associated with “rapid and sustained responses to risk communications.” [8]

We assessed impacts of boxed warnings and REMS on use and adverse outcomes of two drugs (epoetin and darbepoetin)- Erythropoiesis Stimulating Agents (ESAs). These drugs are associated with mortality and venous thromboembolism (VTE) risks when administered to cancer patients [18–28]. ESAs are approved for chemotherapy-induced anemias (CIAs) to minimize transfusions [19,29] Initial trials found a 36% reduction in red blood cell transfusions [18]. Meta-analytic studies found elevated thrombosis risk (7.5% vs. 4.9%) [19], increased mortality risk (Hazard Ratio = 1.10, 95% CI [1.01, 1.20]) [19] and Hazard Ratio = 1.17, 95% CI [1.06, 1.3]) [20] and decreased overall survival when cancer patients received ESAs (Hazard Ratio = 1.06, 95% CI [1.0, 1.12]) [20].

Discovery of adverse events led to boxed warnings in 2007 limiting ESA use to cancer patients with CIA receiving chemotherapy with *palliative* intent. FDA, concerned that label changes were insufficient, mandated in 2010 that ESAs have Risk Evaluation and Mitigation Strategies (REMS) [29,30]. REMS, enhanced regulatory actions [31], included medication guides; communication plans, elements to assure safe use, and plans for monitoring compliance [32].

ESA risk communication chronologies offers opportunities to study safety warnings in diverse settings as shown in Table 1. Past research in non-VA systems found ESA warnings had limited impact [8,20,33–42]. Distinctions between VA and non-VA systems exist [43]. In non-VA settings, drug companies rebate physician offices for administered intravenous drugs [44]. Differences between insurers’ reimbursed prices of intravenously infused biologics and physicians’ rebated prices represents incentives to prescribe *drugs* [44–46]. For VA physicians, financial incentives are lacking. We studied ESAs epoetin and darbepoetin separately, an important distinction [47] because price mark-ups, reimbursement policies, and intensity of pharmaceutical detailing differ between ESAs.

**Table 1:** Chronicle of regulatory actions and safety information related to ESAs.

1989–2002	2003–2006	2007	2010	2017
In 1989, Epogen (epoetin) was approved by the FDA for anemic renal failure patients. In 1993, Procrit (epoetin) was approved for anemic cancer under chemotherapy patients. In 2002, Aranesp (darbepoetin) was approved for both anemic renal failure and anemic cancer patients under chemotherapy.	A majority of evidence from RCTs on increased risks of tumor progression, blood clotting, stroke, heart attacks, and/or mortality associated with the use of ESAs began to emerge.	In March, the FDA issued a black box warning on ESAs used for renal failure and some types of cancer. Immediately thereafter, CMS issued new coverage and reimbursement policies for ESAs.	In March, the FDA issued the ESA REMS for cancer patients with concomitant chemotherapy, mandating medication guides, communication plans, elements to assure safe use, and a monitoring plan.	In April, the FDA removed ESA from the REMS program, citing manufacturer data and additional FDA analyses showing appropriate use of ESAs.

Changing prescribing behavior is essential. Little evidence evaluates warning effectiveness and FDA-mandated REMS in reducing serious adverse events [48–51]. Impacts of warnings and REMS on utilization of ESAs and on adverse outcomes were assessed. Prescribing changes are particularly important in the setting of ESAs, where 2019 guidelines from the American Society of Clinical Oncology/the American Society of Hematology state that “depending on clinical circumstances, ESAs may be offered to patients with chemotherapy-associated anemia whose cancer treatment is not curative in intent and whose hemoglobin has declined to < 10 grams/deciliter [55]. Patterns of care data from South Carolina Medicaid showed that in 2010, 3% and 7% of cancer patients with chemotherapy-induced anemia received epoetin and darbepoetin, respectively [47]. It is not known what the frequency of use of epoetin and darbepoetin in the Veterans Administration system was in 2010- an observation that would have implications for current practice. In particular, if epoetin and darbepoetin use in 2010 in the VA setting were negligible, then this would imply that in the VA setting, clinicians strongly viewed red blood cell transfusion or allowing patients to have lower baseline hemoglobin levels as preferred to administered epoetin or darbepoetin for treatment of chemotherapy-induced anemia.

## Methods

### Data

Data were from VA Integrated Service Networks (VISNs), using the VINCI (VA informatics and computing infrastructure) research environment. The Veterans Affairs Central Cancer Registry (VACCR) includes information on cancer diagnosis date, cancer site, stage of disease, and age, gender, race and ethnicity. VA Pharmacy Benefits files included national drug codes (NDC), prescription date, quantity, VA station number, and physician ID numbers. All of the patient information was anonymized during the data analysis stage. The study was reviewed by the full Institutional Review Board of the WJB Dorn Veterans Administration Medical Center (IRBNet Number: 1007797). The IRB waived the requirement of informed consent.

#### Eligible chemotherapy-treated cancer patient identification

Non-small cell lung cancer, colorectal cancers, and breast cancer were focused on. These cancers differ from each other in patient profiles, clinical characteristics, treatment, and prognoses. All patients had a diagnosis of cancer between 2005 and 2012. Eligible patients were at least 18 years old and younger than 90 years of age. Patients were tracked from chemotherapy administration for as long as chemotherapy was continually administered or death.

#### Chemotherapy administration

Chemotherapy administration was determined from inpatient/outpatient encounter records, pharmacy records, carrier claims and registry data between the date of the diagnosis (for registry cases) or first cancer claim (other cases) and six months later.

### Outcomes

#### ESA treatment use and transfusion

For each course of chemotherapy administration, it was determined whether either epoetin or darbepoetin was dispensed. Use of ESA (epoetin, NDC (national drug code) 55513-126-xx and darbepoetin NDC code 55513-021-xx) was ascertained. Blood transfusion was identified from outpatient encounter data (CPT code 36430–36460).

#### VTE occurrence and mortality

A VTE event required an ICD-9 code in the first or second position on claims related files (the first claim), as per the method of Hershman et al in evaluating ESAs in the SEER-Medicare dataset [52]. For each chemotherapy course, the last epoetin or darbepoetin administration was identified and created an indicator variable equal to 1 for those with a VTE within 60 days after last ESA administration. Two indicators, *vte60epo* and *vte60darbe*, were created. If a patient died within courses of chemotherapy administration, an event observable based on date of death in vital statistics files, an indicator variable “died” was created equal to 1.

#### Chronic conditions, hematocrit and cancer stage

Comorbid disease was based on the Klabunde adaptation of Charlson indices [53]. Medicare inpatient/outpatient codes were searched for ICD-9 diagnostic codes and chronic conditions files. Each condition was weighted. Patients were assigned scores based on the Klabunde-Charlson index given comorbid conditions during chemotherapy administration periods. Hematocrit values were measured as percentages based on the average hematocrit values for three-months prior to ESA initiation. Cancer stage was designated as the closest in time prior to ESA initiation.

### Control variables

#### Sociodemographics

Patient sociodemographic information included age, gender, race/ ethnicity, and marital status. Age was measured from date of birth to date of cancer diagnosis or first claim for cancer. Age was categorized into five-year intervals. Race/ethnicity was categorized as African American, Hispanic, non-Hispanic white, and all others. Patients were identified as married/not.

#### Geographic variables

All data sets except SEER-Medicare provided patient residence at zip code levels. To ensure consistency, the geographic variable was defined at the county level.

#### VA station ID variables

These variables served as fixed effects in segmented regression analyses. These fixed effects absorb time-invariant unobserved characteristics in our longitudinal dataset, such as organizational incentives to prescribe drugs.

### Statistical analyses: Segmented regression analyses

We used segmented linear regression to estimate changes in ESA use changes on levels and trends, clinical appropriateness, and adverse events (VTEs) among chemotherapy-treated cancer patients. The analysis was conducted separately for epoetin only, darbepoetin only, and for both epoetin/darbepoetin. These analyses accounted for baseline level and drug use trends while estimating changes in level and trend resulting from policy interventions. The models included a variable reflecting the number of intervals after the first quarter *(X*_*1*_*(t))*. To estimate changes in level of drug prescription rate after policy actions, we included model-specific indicator variables as covariates based on specific actions were included.

We used slope and trend coefficients to help estimate mean differences between ESA rates of use between the intervention (the observed rate) and times without intervention (the expected rates) for specific quarters. The quarters were based on policy changes and other interventions of interest. The following quarters were evaluated: For ESAs, the first quarter after the FDA-disseminated public health advisories and the concomitant manufacturer-issued boxed warning on the ESA products (April to June 2007), the last quarter after the public health advisories and the box warnings and before initiation of the REMS program (October to December 2009), and the last quarter after REMS (October to December 2016). To assess model assumptions, we tested for residual autocorrelation at various lags. We used, partial autocorrelation and inverse autocorrelation plots to visually assess for residual autocorrelation. The regression had the following specification:

$$y_{ijt}=\beta_{0}+\beta_{1}∙{counter}_{t}+\beta_{2}∙{post\;boxed\;warning}_{t}+\beta_{3}∙{quarters\;since\;boxed\;warning}_{t}+\beta_{4}∙{post\;REMS}_{t}+\beta_{5}∙q{uarters\;since\;REMS}_{t}+\beta_{l}X_{ijt}+\alpha_{j}+\varepsilon_{ijt}$$ (1)

The subscript *i* denotes a unique patient, *j*, a unique VA station, and *t*, the time (in quarter) when chemotherapy began. *Y* is the various outcomes, including ESA use, adverse outcomes (VTE or death), control variables (i.e., Charlson index, hematocrit values, anemia diagnosis, and cancer stage). *Counter* is a variable that counted the number of quarters since the beginning study period (2005 quarter 1). It represented the slope of the trend in the outcome variable before any risk communications. *Post boxed warning* identified the level change in the outcome variable after warnings were announced in 2007 quarter 1. *Quarters since the public health advisories and the boxed warning* is a second counter variable that is 0 in all quarters prior to 2007 quarter 1 and begins counting at 1 through each passing quarter from 2007 quarter 1. The variable captured slope changes from pre-public health notifications and boxed warning periods to post-public health advisories and warning periods. Likewise, *post REMS* and *quarters since REMS* respectively captured the level change and the slope change (from the pre-REMS period) in outcome variables. *X*, a vector of control variables, includes age, race, type of cancer, cancer stage, Charlson index, anemia diagnosis, and hematocrit. However, when one of these control variables was the dependent variable, it was removed as a control variable. Finally, α (VA station fixed effects) controlled for time-invariant VA station-specific heterogeneity, and ε was a random error term.

P-values for coefficient estimates for individual datasets and 95% confidence intervals are reported. All p-values were two-sided with a threshold of 0.05. All analyses were performed using Stata Version 15, with data assembly and cleaning in SQL and SAS.

## Results

**Fig 1:**
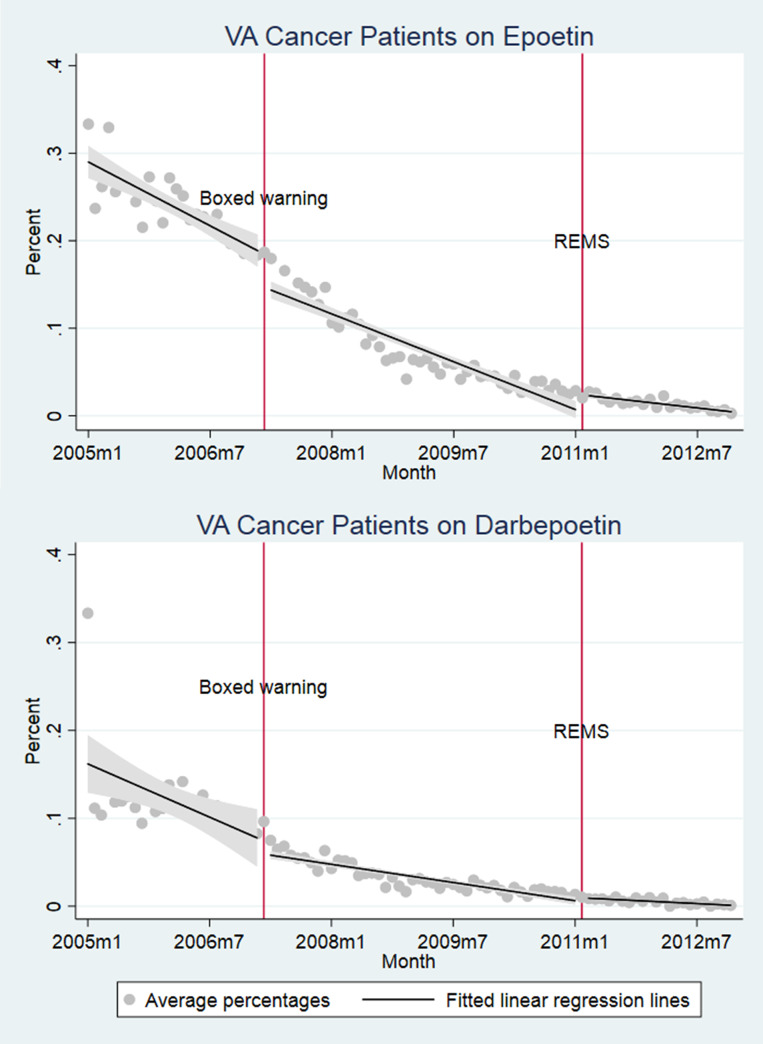
(a) VA cancer patients on epoetin and (b) VA Cancer patients on darbepoetin.

**Fig 2:**
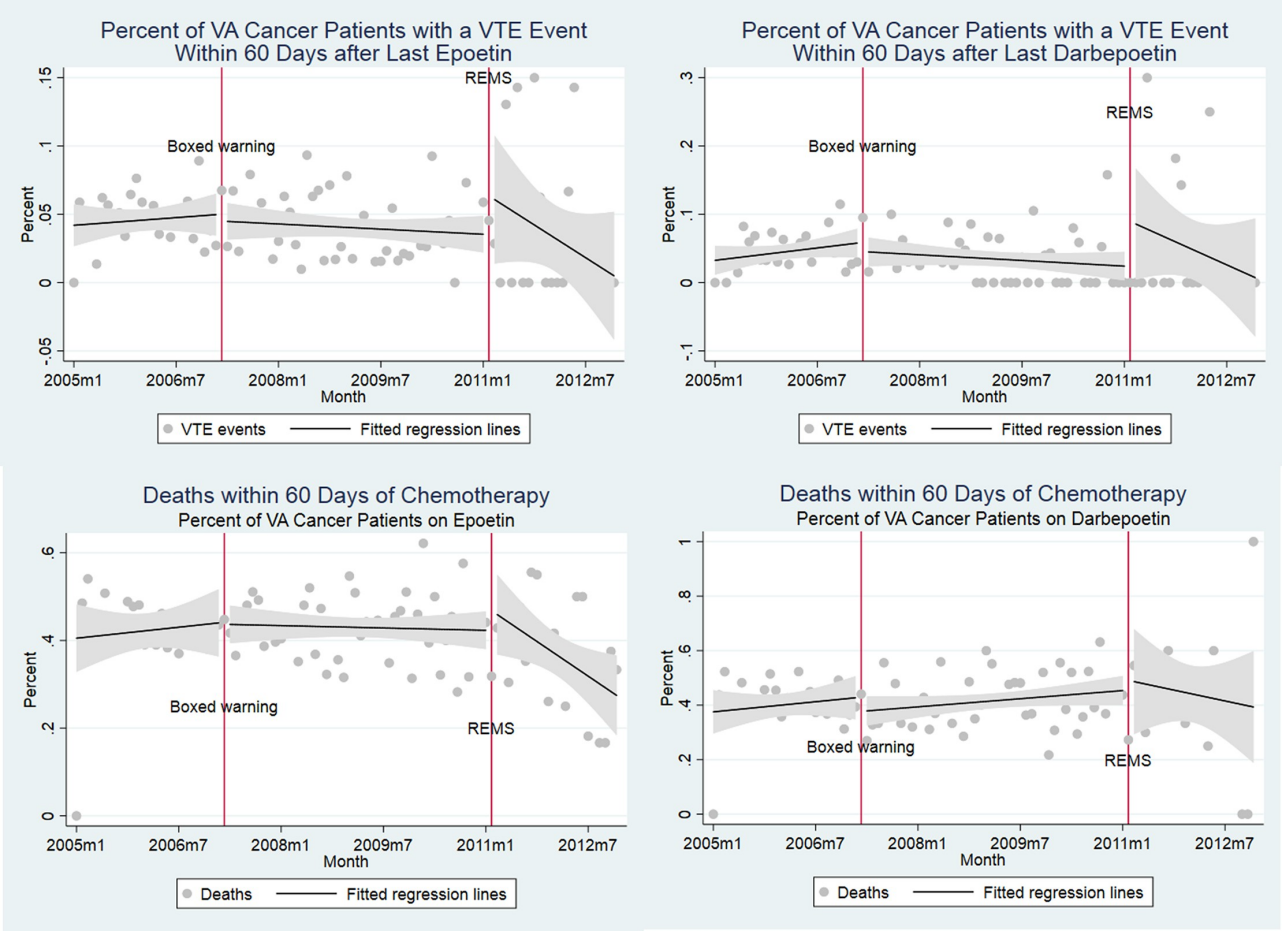
Percent of VA cancer patients within 60 days (a) VTE event after epoetin, (b) VTE event after darbepoetin, (c) death after chemotherapy—epoetin and (d) death after chemotherapy—darbepoetin.

A total of 91,233 courses of chemotherapy administration was identified, some individuals received more than one chemotherapy course (Table 2). Only 8.5% of cancer patients with concomitant chemotherapy received at least one dose of epoetin, and the corresponding percentage for darbepoetin is 3.8%. At the height of ESA use, which occurred before the 2008 Warning, 23.2% and 11% of chemotherapy patients were respectively administered epoetin and darbepoetin (Figs 1 and 2).

**Table 2:** Summary statistics.

	All chemotherapy patients		Chemotherapy and epoetin		Chemotherapy and darbepoetin	
	Mean	St. Dev.	Mean	St. Dev.	Mean	St. Dev.
**N**	73,703		7,051		3,209	
**Age**	67.168	10.076	66.828	9.784	66.677	9.740
**Female (%)**	0.065		0.049		0.049	
**Married (%)**	0.460		0.490		0.487	
**White (%)**	0.649		0.592		0.569	
**Black (%)**	0.123		0.136		0.161	
**Asian (%)**	0.002		0.001		0.001	
**Hispanic (%)**	0.008		0.012		0.004	
**Other race (%)**	0.219		0.259		0.265	
**Epoetin (%)**	0.085		1.000		1.000	
**Darbepoetin (%)**	0.038		0.451		1.000	
**Chemotherapy (%)**	1.000		1.000		1.000	
**Duration (months)**	0.924	1.413	0.390	0.794	0.372	0.757
**Lung cancer (%)**	0.592		0.664		0.682	
**Breast cancer (%)**	0.050		0.028		0.028	
**Colon cancer (%)**	0.254		0.171		0.155	
**Lymphoma (%)**	0.114		0.143		0.140	
**Cancer stage 1 (%)**	0.258		0.146		0.138	
**Cancer stage 2 (%)**	0.375		0.360		0.351	
**Cancer stages 3 & 4 (%)**	0.367		0.494		0.511	
**Charlson Index**	5.499		6.306		6.321	
**Anemia Diagnosis (%)**	0.242		0.659		0.631	
**Transfusion (%)**	0.055		0.214		0.234	
**Died (%)**	0.286		0.433		0.414	
**Hematocrit**	36.883	5.778	33.543	5.484	33.560	5.344

In the overall sample, mean patient age was 67 years, of whom 6.5% were female. Our cohort is 64.9% White, and 12.3% African American. Lung cancer represented the largest percent at 59.2%, followed by colon cancer, and breast cancer at 25.4% and 5%. Patients had advanced cancer- 74.2% at stages 2, 3, and 4. Average Charlson index scores were 5.499, 24.2% of patients had anemia, and 5.5% of patients required blood transfusions. Overall, 28.6% of patients died.

**Fig 3:**
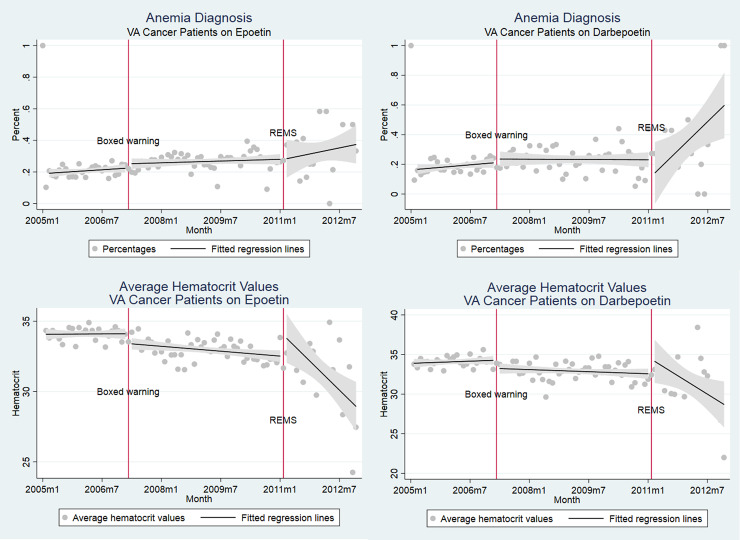
Percent of VA cancer patients with anemia diagnosis (a) epoetin, (b) darbepoetin, and average hematocrit levels of VA cancer patients (a) epoetin, (b) darbepoetin.

**Fig 4:**
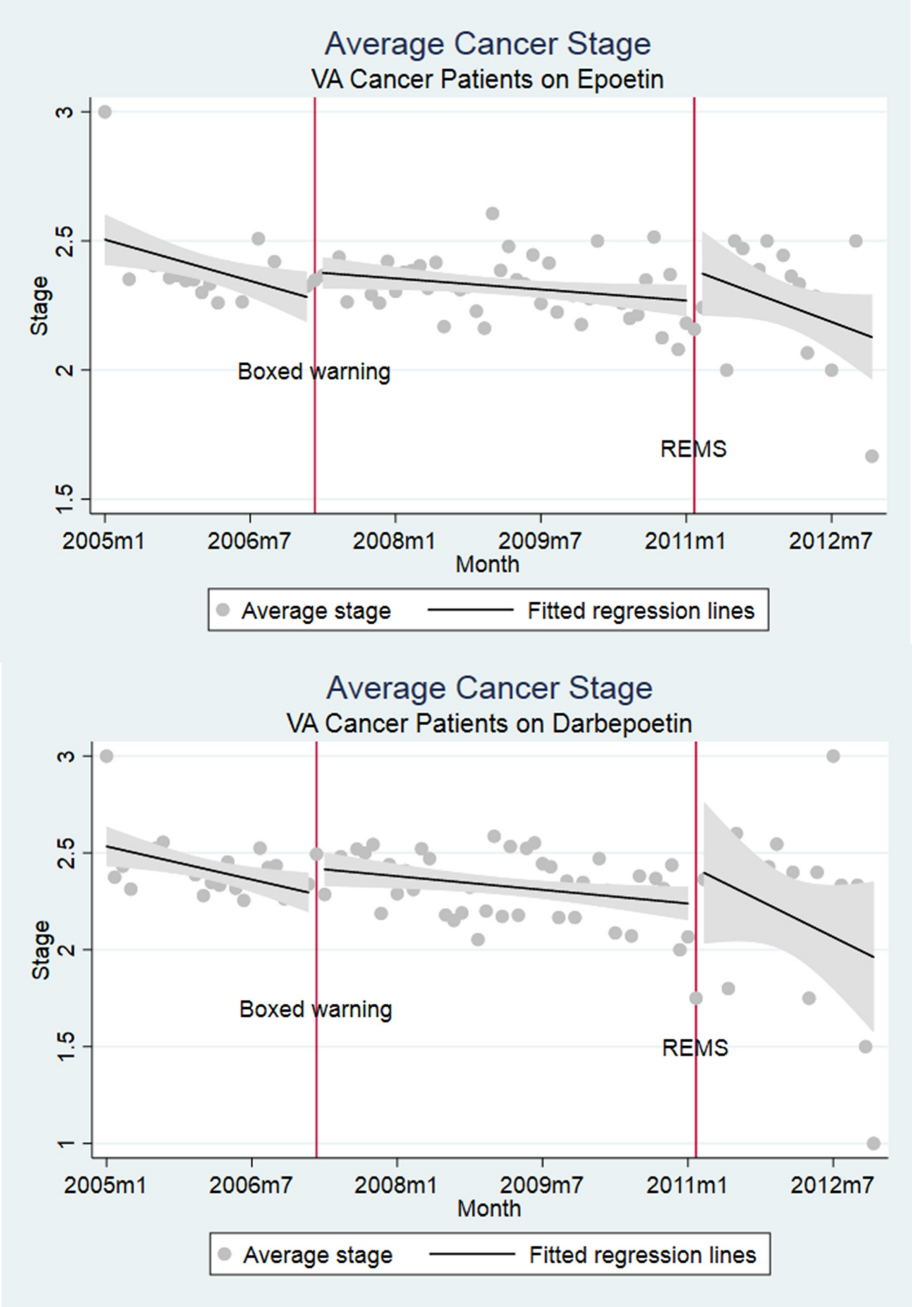
Average cancer state of VA cancer patients for (a) epoetin and (b) darbepoetin.

Cancer patients administered chemotherapy and epoetin or darbepoetin were sicker than chemotherapy only populations, with greater proportions of cancer stages III or IV (49.4% and 51.1%), higher Charlson scores (6.31 and 6.32), greater anemia rates (65.9% and 63.1%) and transfusion (21.4% and 23.4%), and mortality rates (43.3% and 41.4%) respectively for epoetin and darbepoetin users. ESA use dropped after the Boxed Warning, but was falling even prior to this (Fig 1A and 1B). VTE rates and deaths did not inflect at the point of boxed warnings. Clinical indicators coincided with practices counseled by boxed warnings (Figs 3 and 4).

Multivariate regression analyses confirmed three trends: ESA use, both for epoetin and darbepoetin, fell consistently throughout the entire study period (2005–2012), with additional level changes occurring around the time of the boxed warnings; there is no evidence that the warnings reduced adverse events (VTEs and death); and following boxed warnings, VA physicians administered ESAs to patients with higher cancer stage.

The most consistent results are that the boxed warnings were associated with most dramatic reductions in use of epoetin and darbepoetin (S1 Table). The negative coefficients on pre-Boxed Warning trends show that ESA use was already falling prior to the warnings. There were statistically significant discontinuities in ESA use, with reductions of 4.3% (p<0.01) and 3.1% (p<0.01) in likelihood of epoetin and darbepoetin prescription, respectively. Coefficients on changes in trend after the boxed warning, level changes after REMS, and trend changes after REMS are all positive and significant.

Because the cohort consists of all cancer patients on chemotherapy (S2 Table), the remaining regression results are interpreted as temporal trends in outcomes for VA cancer patients on chemotherapy. These results suggest no temporal changes in VTE or mortality risks. There was an increase of 3.7% in the likelihood of having an anemia diagnosis after REMS and increases in average Charlson scores by 0.16 (p<0.01) and 0.32 (p<0.01) after the boxed warning and REMS. The likelihood of being diagnosed as cancer stage 1 increased by 1.8% post Boxed Warning. S2 and S3 Tables focus on trends in outcome variables conditional respectively on epoetin and darbepoetin prescription, respectively. As in regressions using the entire cohort, our results show that neither mortality nor VTE rates fell over time. The most significant result is that hematocrit values at the time of ESA initiation dropped after REMS were initiated (p<0.01 for epoetin and darbepoetin).

## Discussion

Our main findings are ESA use fell consistently throughout the study period, for both epoetin and darbepoetin, but the boxed warnings created an additional reduction in ESA utilization; adverse events (VTEs and death) did not decrease following boxed warnings or REMS; VA physicians responded differentially to the REMS versus the boxed warnings by decreasing the initial hematocrit level when initiating ESAs; and as of 2010, administration of epoetin or darbepoetin for treatment of chemotherapy-induced anemia was negligible.

Our results differ from literature where physicians are reimbursed for administering drugs intravenously in clinical settings. The divergence is interesting, given that all studies assess impacts of boxed warnings on the same drugs in different contexts–the VA system, Medicaid, Medicare, and commercial health insurance. Oure results suggest that financial considerations do not appear to affect how VA physicians react to safety warnings.

In a prior study of the South Carolina Medicaid program where financial considerations are relevant, Noxon et al found differential utilization patterns for epoetin versus darbepoetin following warnings and safety actions [47]. For epoetin, utilization decreased steadily between 2002 and 2010, where darbepoetin use increased between 2003 and 2007 and then decreased thereafter. Per-patient dosing of darbepoetin, but not epoetin, increased between 2003 and 2010, and monthly per-patient epoetin costs decreased 3% while per-patients costs of darbepoetin increased 30% between 2003 and 2010. Noxon et al reported that in 2010, epoetin and darbepoetin were administer to 3% and 7%, respectively, or cancer patients with chemotherapy-induced anemia [54]. In contrast, in the VA system in 2010, 1% of cancer patients with chemotherapy-induced anemia received epoetin or darbepoetin, respectively- strongly indicating the “end of the ESA era” at least in the VA system.

Among Medicare providers where financial considerations for individual providers are relevant, Bian et al showed that compared with a control group, ESA use started declining sharply after warnings were issued, concomitant with Center for Medicare and Medicaid Services (CMS) institution of a National Coverage Decision mandating non-payment for ESAs if hematocrit levels were > 30%, whereas ESA use trends remained similar with the control before warnings [55]. VTE trends were stable. The study found that the boxed warnings/CMS decision were associated with a 20.2-percentage-point reduction in likelihood of ESAs being used in the cancer setting, but were not associated with VTE reductions.

Our study contradicts with results from a study by Schoen et al that uses the IMS LifeLink™ Health Plan Claims Database, which is composed of commercial health plan information [56]. They found that odds of receiving epoetin decreased after risk communications and REMS, but initially increased for darbepoetin after box warnings were disseminated. They found that the odds of receiving a high darbepoetin dose increased from 2001 to 2012, whereas odds of receiving a high epoetin dose decreased after REMS, suggesting that physicians increased per-patient darbepoetin dosing as the prevalence of darbepoetin use declined. Also, epoetin and darbepoetin rates of use in 2012 for chemotherapy-induced anemia were 3.1% and 2.6%, respectively, while in the VA, these rates of use were 1% and 1%, respectively.

Our study findings can be compared to those reported for Ontario, Canada for ESA use among cancer patients with chemotherapy-induced anemia between 2007 and 2009 and for VA use between 2002 and 2008 [57,58]. Tarlov et al. and our study identifies the beginning of marked decrease in ESA use for chemotherapy-induced anemia beginning in 2005, following publications of poorer survival and high venous thromboembolism rates among ESA-treated cancer patients [57]. Their data is limited by not following ESA use beyond 2009 prior to initiating the ESA REMS program. Weir et al report that in Ontario, ESA use for chemotherapy induced anemia did not begin to decline until Canada Health and the FDA disseminated Black Box warnings about ESA use in these settings in 2007 [58]. In Canada, ESA use appeared to be influenced by national policies and regulatory decisions, whereas VA use of ESAs is strongly influenced by physician practice patterns, which changed markedly following publication of two large randomized controlled trials with ESAs in 2003.

While other incentive-driven contexts showed divergent utilization patterns between epoetin and darbepoetin, this was not seen herein. Yet ESA suppliers likely marketed their drugs with different degrees of aggressiveness. Amgen, the supplier of darbepoetin, settled civil litigation for allegations of over-promotion and over-marketing of darbepoetin (for $610 million to the Department of Justice and $72 million to 48 state Medicaid programs) and pled guilty to related criminal charges (paying $160 million- the largest federal fines/settlements in U.S. history related to a biologic product)) [47]. VA physicians, who are paid a fixed salary, were less susceptible to pharmaceutical detailing.

## Conclusion

ESA use fell in the VA system from 2005, even prior to the 2007 FDA boxed warnings for epoetin and darbepoetin, a finding that was initially reported by Tarlov et al. when evaluating VA use of ESAs between 2002 and 2009 [57]. Boxed warnings served as additional nudges to reduce utilization following safety-related concerns identified in clinical trials in 2003 and conform to clinical suggestions in warnings, such as to advise ESA use only among cancer patients treated for palliative intent. Possibly due in part to the low ESA use 2010, we did not find significant reductions in adverse outcomes because variance around these outcomes was great. We also found that the mean hematocrit level decreased following the 2010 REMS—indicating that patient consent may have been associated with patient and physician reluctance to receive ESAs at higher hematocrit levels. Our results suggest that boxed warnings had intended effects in the VA, contrary to other insurance contexts where financial incentives to prescribe profitable injectable drugs may distort impacts of risk communications. REMS provided additional disincentives against ESA use that were not noted following the boxed warnings.

Taken in context with other studies by Bian et al. [55] for Medicare, Weir et al. [58] for Ontario, Canada, Noxon et al. [54] for South Carolina Medicaid, the results suggest that safety actions may have differential reactions across insurance types. ESA use appeared to have fallen earlier and was more consistent for epoetin and darbepoetin in the VA than in systems with financial incentives. This finding can help policymakers tailor messaging and supporting policies and/or regulatory action to specific insurance contexts. Policymakers may consider measures such as tort law changes (to raise “cost” of not heeding FDA risk communications), drug reimbursement policy reform and legislative reform on pharmaceutical marketing and detailing (to lower overprescribing), including a more nuanced interpretation of First Amendment free speech rights for off-label marketing.

A final important conclusion is that in the VA system, the era of administering epoetin or darbepoetin to cancer patients with chemotherapy-induced anemia appeared to end in 2012- a finding that is different from that reported among cancer patients covered by private health insurance or Medicaid health insurance.

## Supporting information

S1 Table Segmented regression analysis (all chemotherapy patients). (DOCX)

S2 Table Segmented regression analysis (epoetin). (DOCX)

S3 Table Segmented regression analysis (darbepoetin). (DOCX)
