# Supplementary material for: End of an era of administering erythropoiesis stimulating agents among Veterans Administration cancer patients with chemotherapy-induced anemia
Source: PLoS One. 2020 Jun 25;15(6):e0234541. doi: 10.1371/journal.pone.0234541 (PMC7316310; doi:10.1371/journal.pone.0234541)
Supplement: S2 Table — (DOCX) [file pone.0234541.s002.docx]

**Table S2: Segmented Regression Analysis (Epoetin)**
